# Neurodevelopmental disorders in children with cleft lip and palate: a systematic review

**DOI:** 10.1007/s00787-024-02636-y

**Published:** 2024-12-26

**Authors:** Benjamin Cook, Stef Van Bockstaele, Samuel B. Crow, David Sainsbury, Sophie Butterworth, Simon Filson

**Affiliations:** 1 Faculty of Life Science and Medicine, King’s College London, London, UK; 2 Northern and Yorkshire Cleft Lip and Palate Service, Newcastle Upon Tyne NHS Hospitals Trust, Newcastle, UK; 3 Department of Plastic Surgery, Evelina Hospital, St Thomas’ Hospital, London, UK

**Keywords:** Cleft lip and palate, Orofacial clefts, 22q11 deletion syndrome, Attention deficit hyperactivity disorder, Autism spectrum disorder

## Abstract

Individuals with orofacial clefts (OFCs) may be at an increased risk of developing autism spectrum disorder (ASD) and attention deficit hyperactivity disorder (ADHD). This systematic review provides a summary of the most recent data regarding the prevalence of ASD and ADHD in the OFC population and compares this to the general paediatric population. Multiple databases were searched including PubMed/Medline and Embase in July 2024, following the Preferred Reporting Items for Systematic Reviews and Meta-analyses guidelines and was registered in PROSPERO (CRD42024565219). 1025 papers were identified for screening, of which nine were included in the report. Percentage prevalence was calculated and compared to global prevalence or control populations where available. Overall, ASD prevalence among children with a cleft ranged from 0 to 50% (Mean = 2.87%; SD = 5.40) compared to ~ 1% globally (WHO) and ADHD prevalence ranged from 2.34 to 31.71% (Mean = 3.63%; SD = 4.30) compared to ~ 5% globally (NICE). Matched control populations showed larger differences. Isolated cleft palate was associated with higher rates than isolated cleft lip or combined cleft lip and palate. Prevalence in individuals with syndromic clefts appeared greater still (Mean = 14.80%; SD = 16.58) although populations were small. Children with OFCs demonstrate increased prevalence of ASD compared to the average paediatric population. Evidence for increased prevalence of ADHD is less clear, with varying rates across studies. Children with isolated cleft palate or cleft associated with genetic syndromes appear most at risk, although further research is needed.

## Clinical Relevance and Implications

Literature has demonstrated a potentially elevated risk of certain neurodevelopmental disorders in individuals with orofacial clefts (OFCs). This study found an increased prevalence of ASD in children with orofacial clefts compared to matched controls and global prevalence. ADHD may also be increased in these populations, although data is less clear. Both ASD and ADHD prevalence appeared even greater in individuals with syndrome-associated OFCs, as well as those with cleft palate only (CPO). Clinical assessments should involve greater vigilance for ASD and ADHD amongst both clinicians and parents/guardians through patient education. Future research is required to clarify the nature of the association between ASD/ADHD and OFCs and identify any causative factors that may lead to this increased risk.

## Introduction

Cleft lip and/or palate is a congenital craniofacial malformation which affects around 1 in 700 births in the UK [1], and around 195,000 children are born worldwide with an orofacial cleft (OFC) each year [2]. Clefts are typically evident from birth, with neonates potentially showing changes in facial appearance and symptoms of respiratory distress and reflux during feeding, prolonged feeding time and infant fatigue. Physical examination will reveal a palatal cleft, except in the case of submucosal cleft [3].

Classification of OFCs typically differentiates either the aetiology of disease, or the anatomical changes associated with each cleft sub-type. OFCs can be either syndromic or non-syndromic/idiopathic (i.e. not associated with an underlying genetic disorder). However, the exact aetiology of idiopathic clefts may indeed include genetic factors, along with others such as differences in environment, family history and race [4]. Syndromic clefts form a smaller percentage of total OFC cases (5–7%) [5], yet there are numerous genetic syndromes causing clefts including: 22q11.2 microdeletion syndrome (22q11DS, velocardiofacial syndrome, Di George syndrome); Van der Woude’s syndrome, Stickler’s syndrome and Treacher Collins syndrome [6]. Further classification can be carried out through distinction between isolated cleft palate (CPO), isolated cleft lip (CL), and combined cleft lip and palate (CLP), as well as between unilateral and bilateral clefts.

Among children with OFCs, numerous studies have noted higher rates of neurodevelopmental and psychosocial disorders compared to the paediatric population without a cleft. A recent study demonstrated nearly 1 in 10 children with craniofacial abnormalities (including a cleft) had a diagnosis of ASD; significantly higher than the control population [7]. However, prevalence has varied significantly between studies, as have diagnostic methods. This systematic review synthesises the current available data on ASD and ADHD prevalence in the population of children with OFCs, including those with or without an associated syndrome.

## Materials and methods

### Search strategy

**Fig. 1:**
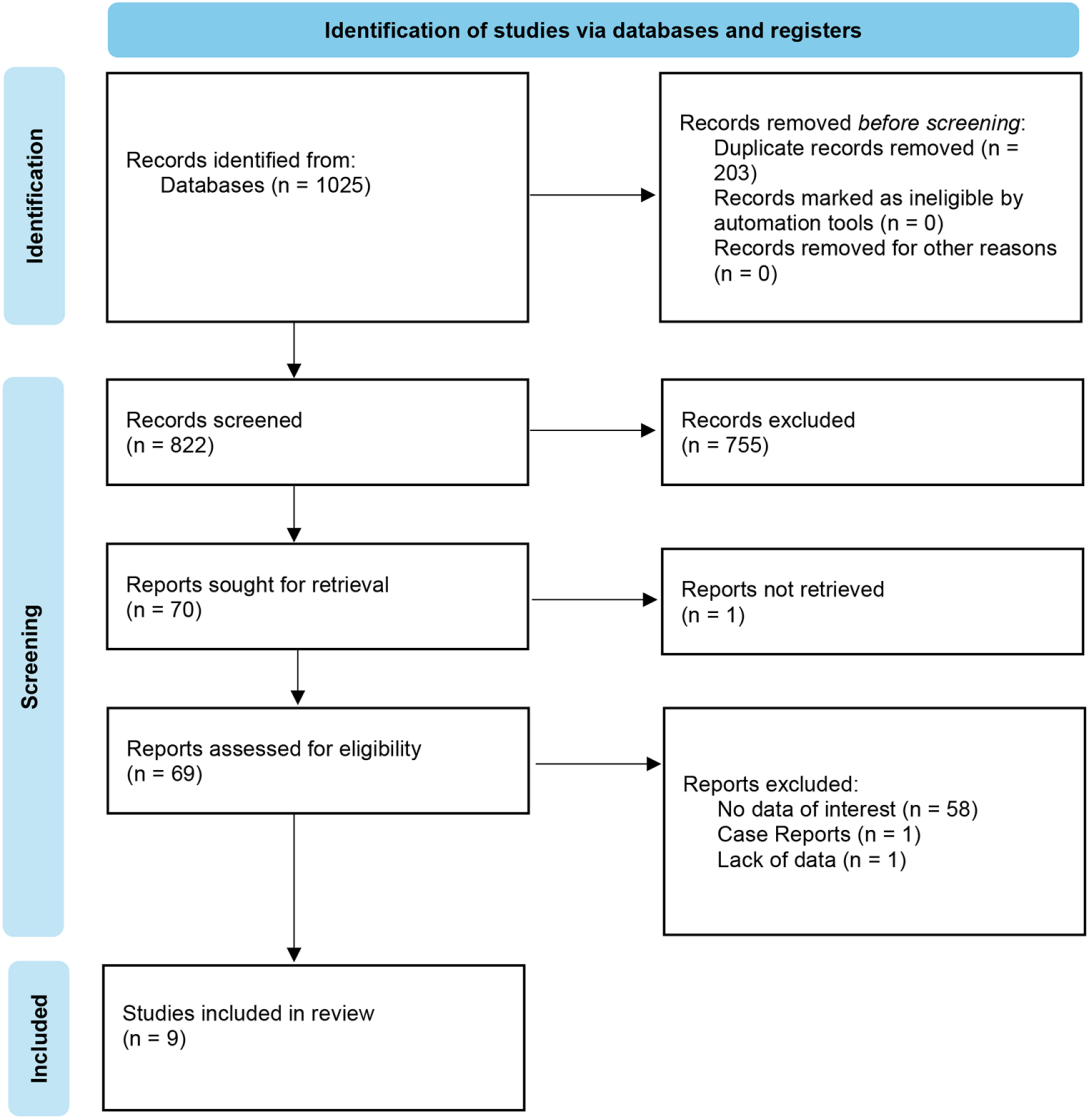
PRISMA flow diagram. PRISMA = Preferred Reporting Items for Systematic reviews and Meta-analyses

This review followed the Preferred Reporting Items for Systematic Reviews and Meta-Analyses (PRISMA) methodology [8], and the review protocol was registered on PROSPERO (CRD42024565219). Figure 1 outlines the strategy used. PubMed/MEDLINE and Embase were searched in July 2024 using the following search string: (“Cleft palate” OR “Cleft lip”) AND (“ADHD” OR “ASD” OR “Autism”) to identify articles published within the last 100 years. Articles in English, or those where a translated article was available, were included.

### Review of studies

The initial search heralded 1025 articles across both databases. Duplicates were removed, facilitated by the software EndNote, with 822 articles selected for screening. Abstract only articles were included due to the sparsity of relevant manuscripts. Articles were individually screened, retrieved and reviewed by two reviewers (B.C. and S.V.B.). Exclusion criteria were as follows: (1) Duplicates identified manually, (2) No data of interest, (3) Case reports, (4) Lack of data (i.e. not enough information was given for data analysis). Nine studies were eventually selected for inclusion in the review. The bibliographies of included studies were reviewed, but no further data of importance or relevant articles were found. Disagreements between reviewers were dealt with at the discretion of the supervising authors (S.F. and D.S.). Further to the initial search, an additional reviewer (S.C.) reassessed included reports for eligibility. No changes were subsequently made.

### Data extraction

Data extraction was initially carried out by one of the review authors (B.C.) with a second review author (S.C.) cross referencing extracted data to the original articles. The following data was extracted and included in the review: (1) Total population with an OFCs i.e. CL + CLP + CPO, (2) Total control population where available, (3) Number of ASD/ADHD diagnoses in the population with an OFC, (4) Number of ASD/ADHD diagnoses in the control population. Percentage prevalence per study (ASD/ADHD diagnoses / n) was calculated. Studies not including a control population were compared to nationally available prevalence figures. Only ASD or ADHD diagnosed through neuropsychological assessment were included in the review. Further analysis between different population characteristics (e.g. age) was not carried out but was discussed where available. An exception to this is the distinction between cleft types – CL/CLP/CPO differentiation and non-syndromic vs. syndromic (22q11.2DS) clefts were analysed separately.

## Results

Nine studies were analysed, and their data extracted (Table 1). Data across studies varied significantly, as did population size; therefore, weighted means (M) and standard deviation (SD) were calculated. Across all studies, rates of ASD in the OFC population ranged between 0% and 50% (M = 2.87%; SD = 5.40), compared to ~ 1% globally (WHO) [9], suggesting a rate two to three times higher among people with a cleft. Prevalence of ADHD ranged between 2.34% and 31.71% (M = 3.63%; SD = 4.30) and reflected rates similar to the global figures of ~ 5% (NICE) [10]. However, control populations in the included studies often demonstrated ADHD rates below that of the population with an OFC. Analysis of cleft subtypes showed those with CPO had the highest rates of both ASD (3.41%) and ADHD (3.67%) compared to CL or CLP. Similarly, syndromic OFCs had ASD rates far in excess of non-syndromic OFCs: (M = 14.80%; SD = 16.58) versus (M = 2.02%; SD = 0.87) and ADHD followed the same trend: (M = 25.76%; SD = 13.86) versus (M = 3.03%; SD = 0.36). However, populations in the syndromic studies were far smaller.

**Table 1:** Prevalence of ADHD and ASD in OFCs

Study	Total number with an OFC	ASD (*n*)	ADHD (*n*)	Total number of controls	ASD (*n*)	ADHD (*n*)
**Non-syndromic**	**9673**					
*Huang et al.*	1158	0.35% (4)	3.97% (46)	11,580	0.06% (7)	0.55% (64)
*Tillman et al.*	7842	2.44% (191)	2.89% (227)	78,409	0.98% (765)	2.04% (1839)
	(CL) 2526	1.46% (37)	2.34% (59)	25,253	0.97% (246)	2.10% (530)
	(CPO) 3436	3.41% (117)	3.67% (126)	34,356	0.96% (330)	1.90% (653)
	(CLP) 3048	1.90% (58)	2.66% (81)	30,476	1.01% (309)	2.15% (656)
*Junaid et al.*	673	0% (0)				
**Syndromic**	**733**					
*Fine et al.*	98	14.29% (14)				
*Vorstman et al.*	60	50.00% (30)	6.67% (4)			
*Antshel et al.*	84		42.68% (36)	38		26.32% (10)
*Antshel et al.*	41	41.46% (17)				
*Niklasson et al.*	100	30.00% (30)	23.00% (23)			
*Angkustsiri et al.*	29	17.24% (5)				
*Junaid et al.*	361	1.66% (6)				

*Abbreviations*. ASD, Autism Spectrum Disorder; ADHD, Attention Deficit Hyperactivity Disorder; OFC, Orofacial Cleft; CL, Cleft Lip; CPO, Cleft Palate Only; CLP, Cleft Lip and Palate

### Non-syndromic OFCs

Huang et al. analysed the specialised databases of behavioural health and special health care conditions of Taiwan NHIRD to investigate the prevalence of ASD and ADHD (among other neurodevelopmental disorders) in children with OFCs [11]. This database recorded ‘severe congenital abnormalities’ diagnosed between 2000 and 2011 – in the case of OFCs describing those requiring multiple plastic surgeries and language rehabilitation. Formal diagnoses of CPO, CL and CLP were guided by the International Classification of Diseases, Ninth Revision, Clinical Modification (ICD-9-CM: 479.0, 479.1 and 479.2 respectively) and reviewed by a panel of experts (a formality among Taiwanese medical insurance administrations). ICD-9-CM was again used for formal ASD and ADHD diagnoses (299 and 314 respectively). A random cohort of 3,000,000 individuals from the Taiwanese Longitudinal Health Insurance Database were used for identification of controls groups, matched 1:10 across age, sex, residence and income. 1158 children and adolescents with OFCs, along with 11580 age/sex matched non-OFC controls were identified for inclusion. Of those with OFCs, 499 (43.1%) had CPO, 25 (2.2%) had CL and 634 (54.7%) had CLP. Prevalence of ADHD in patients with OFCs were greatly increased compared to the control population (3.97% vs 0.55%). ASD continued this trend, albeit to a lesser extent (0.35% vs 0.06%).

A larger study by Tillman et al. analysed data obtained from the National Board of Health and Welfare in Sweden as well as Statistics Sweden, to identify 7,900 children born with an OFC between January 1st, 1973 and December 31st, 2012 [12]. Again, controls (1:10) were matched for age, sex and country of birth. Patients with comorbid congenital malformations or other indicators of a syndromic cause of the OFC were excluded. ICD 8, 9 and 10 were used for formal OFC diagnoses (CL, CPO and CLP). ICD 9 and 10 guided ASD and ADHD diagnoses. In total, 7,842 children with OFCs were included, along with 78,409 matched controls. Population breakdown by cleft subtype was as follows: 2,526 (32%) CL; 3,048 (39%) CLP; 3,436 (43%) CPO – Note, this population sums to greater than the total OFC population stated, but it is unclear why. Data analysis focuses on these groups separately but calculation of total prevalence among OFCs will use the reported total population Fig. (7842). Overall, 191 children with an OFC received a diagnosis of ASD, and 227 received a diagnosis of ADHD. In this cohort, ASD showed an increased prevalence compared to the control population (2.70% vs. 0.98%), with ADHD only showing a modest increase (3.39% vs. 2.04%). Cleft subtypes showed varying prevalence of ASD and ADHD, with CPO demonstrating the highest prevalence of both ASD (3.41%) and ADHD (3.67%).

While not strictly included in this review due to insufficient data presented in the available abstract, a study by Khoshab et al. warrants mention [13]. A single-centre retrospective analysis of the 2018 National Survey of Children’s Health Database identified 619 children with non-syndromic cleft lip and/or palate (NSCLP) and 29,147 controls. Children with CL or CLP showed higher rates of ADHD compared to the national cohort: CL (7.7% versus 2.3%), unilateral CLP (4.6% versus 2.3%), bilateral CLP (5.9% versus 2.3%). Comparatively, among children with CPO, rates of ASD and ADHD appeared comparable to the national cohort (4.3% versus 5.5%, 2.7% versus 2.3%, respectively).

### Syndromic OFCs

In an early 2005 study, Fine et al., investigated a population of 98 children with 22q11DS [14]. Out of the total population, 14 children (14.29%) were subsequently diagnosed with ASD by ADI-R (Autism Diagnostic Interview – Revised) and 20 (20.41%) demonstrated ASD symptoms. The ADI-R informed numerical scores in three key areas, where children meeting the diagnostic criteria in two of the three are given a research diagnosis of ASD guided by the DSM-IV (Diagnostic and Statistical Manual of Mental Disorders, 4th Edition) and ICD-10.

In a 2006 paper, Vorstman et al.. evaluated 60 child and adolescent patients recruited from the parents’ network of 22q11DS Children in The Netherlands [15]. Both ASD and ADHD diagnosis followed the DSM-IV criteria, with formal ASD diagnosis including the use of the ADI-R. Where direct assessment was unavailable, caregiver assessment was used. Of the 60 subjects, 30 (50%) were diagnosed with ASD, and 4 (6.567%) with ADHD (one with inattentive subtype ADHD, the three others with hyperactive/impulsive subtype). Some patients who did not receive a diagnosis of ASD were also noted to have autism symptoms, despite not reaching the cut-off in the three diagnostic domains.

Similarly, Antshel et al. studied the prevalence of psychopathology in 22q11DS [16]. One hundred fifty-four children were recruited from the Center for the Diagnosis, Treatment, and Study of VCFS at SUNY-Upstate Medical University. The K-SADS-PL (Schedule for Affective Disorders and Schizophrenia for School-aged Children) semi structured interview was used to guide DSM-IV ADHD diagnosis. Thirty-six (42.68%) of the 84 children had ADHD, compared to 10 (26.32%) of the community control population. Antshel et al. later investigated 41 children with 22q11DS recruited from the center mentioned previously [17]. The ADI-R was once again used for ASD diagnosis, although it was noted this did not qualify the individual for an autism diagnosis. Seventeen (41.46%) participants met the diagnostic criteria for ASD. Antshel noted that ~ 80% of those with 22q11DS and ASD had a concurrent ADHD diagnosis, over twice the rate among those with just 22q11DS (~ 40%). Exact number of individuals was not given and as such, this data was not included in our analysis.

Niklasson et al. assessed the prevalence of autism and ADHD (among other conditions) in a cohort of 100 individuals – 98 of whom were enrolled in a multidisciplinary study at Queen Silvia Children’s Hospital in Gothenburg, Sweden [18]. ASD and ADHD diagnoses were guided by neuropsychiatric assessment adhering to the DSM-IV symptom criteria (ADI-R or diagnostic interviewing was not used due to time constraints). Twenty-three (23%) children were diagnosed with ASD (+/- ADHD), and 30 (30%) received an ADHD diagnosis (+/- ASD). Nine (9%) individuals were diagnosed with both ASD and ADHD.

A 2015 study by Angkustsiri et al. identified 100 children through recruitment from the Cognitive Analysis and Brain Imaging Laboratory web page or the MIND Institute’s Volunteer Research Registry [19]. Twenty-nine children subsequently underwent ASD testing through both the ADOS (Autism Diagnostic Observation Schedule) and SCQ (Social Communication Questionnaire) clinical assessments (ADOS considered more reliable in ASD diagnosis than SCQ, therefore its results are included [20]). The number of children scoring above the ADOS cut-off for ASD was five (17.24%), with one (3.45%) child achieving a score suggestive of a diagnosis of autism. Only 2 (6.90%) achieved SCQ scores suggestive of ASD and notably no child achieved diagnostic scores in both assessments. A parental report questionnaire (BASC-2 PRS) was also used, this time for all 100 participants of the larger cohort. Of this group, 44 (44%) screened positive on this questionnaire.

Most recently, Junaid et al. investigated the prevalence of both intellectual disability (ID) and ASD in a cohort of 1421 children with craniofacial abnormalities in Western Australia [7]. Of this cohort, 1034 children had an OFC, 673 of which were non-syndromic and 361 were syndromic. ASD was either diagnosed through the DSM-IV or DSM-5 (Diagnostic and Statistical Manual for Mental Disorders, 5th edition) criteria, ascertained through the Disability Services Commission (DSC). However, exact diagnostic process (i.e. ADOS, SCQ, ADI-R interviewing) was not described. Six children (1.66%) were diagnosed with ASD in the syndromic cohort. Contrary to previous studies, no child was diagnosed with ASD in the non-syndromic cohort.

## Discussion

The findings of this review indicate a significant increase in prevalence of ASD among individuals with an OFC when compared globally, or to control cohorts. ADHD painted a less clear picture, with rates similar to global prevalence figures, although matched control populations did show an increase among the OFCs cohort. The increase was observed across both non-syndromic and syndromic populations (to a much greater extent in the syndromic OFC studies), however variations in methodology warrants mention. Larger national or multi-national cohort studies are needed with strict diagnostic processes to ascertain the true prevalence in this population. This is the first systematic review to assess for an association between both syndromic and non-syndromic clefts and both ADHD and ASD, thus there is no prior consensus to compare with.

Variations in the criteria used for ASD diagnosis can make analysis difficult (Table 2). For example, the DSM-IV assesses patients across three categories: (1) impaired social interaction, (2) impaired social communication, (3) restricted behaviour pattern, whereas the DSM-5 assesses across only two: (1) Impairments in social communication and social reciprocity, (2) the presence of restricted interests and repetitive behaviours [21, 22]. There has also been concerns raised about the DSM-5 criteria in numerous aspects including diagnosis in infancy and adulthood. While both these criteria were the most recent at the time of publication, it is possible that differing proportions of patients would have met the threshold for diagnosis, should the ASD diagnostic criteria be standardised across all studies. A similar discrepancy may arise when comparing ICD-9 & 10 criteria to DSM-IV & 5 criteria. Again, assuming standardised criteria across studies, it is possible that prevalence may change in certain populations. The same issue arises when comparing ADHD diagnosis; DSM-5, for example, distinguishes different diagnostic criteria depending on the patients age, which DSM-IV does not. Similarly, DSM-5 requires some symptoms to have been present before age 12 years, compared to before age 7 years in DSM-IV [23].

Heterogeneity of diagnostic methods also complicates analysis of the rates. The ADOS and ADI-R differ significantly in the fact that ADOS involves a direct interactive interview with the child, compared to the retrospective interview with the child’s parent or carer used in the ADI-R. It has been suggested that the ADI-R plays a less significant role in ASD diagnosis than behavioural observation (especially in adolescents and adults); instead, although time consuming, a combination of both should be used to guide complete diagnosis [24]. Unlike with ASD, ADHD has a less clear cut ‘gold standard’ for clinical diagnosis, and instead often is made up of a mixture of clinical assessments, interviews and diagnostic scoring criteria [25]. It is therefore difficult to be critical of the diagnostic methods used in the included studies, although again, methodological differences are clear and may play some role in explaining the discrepancies between rates. Even with strict diagnostic criteria, constricted ranges of facial affect and speech difficulties leading to communicative impairment in ASD may be difficult to distinguish from those caused by clefts or other orofacial defects. Feeding difficulties due to clefts have previously been misdiagnosed as eating disorders [12] - it therefore stands that a similar misdiagnosis may occur for ASD or ADHD.

**Table 2:** Diagnostic criteria

Study	Diagnostic criteria		Diagnostic method	
	ASD	ADHD	ASD	ADHD
*Huang et al.*	ICD-9-CM	ICD-9-CM		
*Tillman et al.*	ICD-9, 10	ICD-9, 10		
*Fine et al.*	DSM-IV/ICD-10		ADI-R	
*Vorstman et al.*	DSM-IV	DSM-IV	ADI-R	Caregiver assessment
*Antshel et al.*		DSM-IV		K-SADS-PL
*Antshel et al.*	DSM-IV		ADI-R	
*Niklasson et al.*	DSM-IV	DSM-IV	Neuropsychiatric assessment	Neuropsychiatric assessment
*Angkustsiri et al.*			ADOS	
*Junaid et al.*	DSM-IV, 5			

*Abbreviations*. ASD, Autism Spectrum Disorder; ADHD, Attention Deficit Hyperactivity Disorder; ICD-9-CM, International Classification of Disease, 9th Revision, Clinical Modification; ICD9, International Classification of Disease, 9th Revision; ICD10, International Classification of Disease, 10th Revision; DSM-IV, Diagnostic and Statistical Manual of Mental Disorders, 4th Edition; ADIR, Autism Diagnostic Interview, Revised; K-SADS-PL, Schedule for Affective Disorders and Schizophrenia for School-aged Children; ADOS, Autism Diagnostic Observation Schedule; DSM-5, Diagnostic and Statistical Manual of Mental Disorders, 5th Edition

Across all studies, age range varied dramatically. Although there is relative diagnostic stability for ASD from as young as 14 months, there is also a high rate of false negatives at younger ages (23.8% receiving a diagnosis at 3–4 years of age had a missed diagnosis at their first assessment) [26]. Thus, it may be difficult to draw confident conclusions from studies with larger age ranges and particularly younger participants, such as Fine et al.. who reported a range from 2 to 12 years [14] and Niklasson et al. with a range from 1 to 35 years [18]. Of note, those reporting larger age ranges noted similar ASD and ADHD rates in the older proportion of their populations compared to the younger ones.

Various potential reasons for an increased risk of ASD/ADHD have been identified in the literature. The use of general anaesthesia in cleft patients has been suggested to play some role, with research suggesting early exposure was associated with significantly increased risk of developing ASD (1.65% vs. 0.98% in the control, *P* < 0.01) [27]. Another potential reason for the association is the early use of antibiotics accompanying the surgeries required by patients with OFCs. A recent cohort study found slightly elevated risk among those receiving antibiotics before 2 months of age (Adjusted HR:1.23, 95% CI 1.17 to 1.28) and with antibiotic use over 15 days [28]. There is some evidence to suggest that risk factors such as the use of alcohol and smoking during pregnancy increase the risk of both ADHD and OFCs in children, although data around this remains controversial [29, 30]. Antshel et al. also found neuroanatomic variations (including an enlarged amygdala) reported in idiopathic ASD that were present in children with both VCFS and ASD [17]. Whilst more recent research is suggestive of variations across broader, interconnected neuroanatomic systems, structural imaging of the brain may still aid future assessment and management of ASD [31]. Each of these factors could provide further avenues for research in determining aetiology, as well as enhancing earlier identification of neurodevelopmental disease and subsequent management to improve patient quality of life. It is possible that a combination of several of these risks with other biopsychosocial influences contribute to the development of ASD and ADHD, thus future clinical interventions may include preventative measures and an emphasis on patient education and awareness for early recognition.

## Conclusion

In conclusion, rates of ASD in both the non-syndromic and syndromic cleft populations appear to be significantly higher than that of matched control populations, or the estimated global prevalence. The increased prevalence of ADHD seems far less compelling, with studies demonstrating significantly varied rates. Compared with matched controls, ADHD follows a similar pattern to ASD with higher rates found among those with an OFC; although when compared to global figures, rates do not show appear to be increased. Notably, individuals with isolated cleft palate or a syndromic cleft appear to be at the greatest risk of being diagnosed with ASD or ADHD. Cleft teams should have a higher clinical suspicion of ASD in children with any type of cleft, and prompt referral should be sought in children demonstrating behavioural or speech challenges suggestive of autism or other related neurodevelopmental disorders. Due to the difficulty of diagnosis, specialist input should be sought from multiple disciplines, and the implementation of neuropsychological assessments should be considered in the routine assessments of children diagnosed with a cleft. Further studies are required to improve the diagnostic accuracy of ASD and ADHD through standardised methodology and provide a clearer picture of the prevalence of neurodevelopmental conditions in children with all forms of OFCs.

## Data Availability

No datasets were generated or analysed during the current study.
